# Sagittal abdominal diameter is a more independent measure compared with waist circumference to predict arterial stiffness in subjects with type 2 diabetes - a prospective observational cohort study

**DOI:** 10.1186/1475-2840-12-55

**Published:** 2013-03-28

**Authors:** Elsa M Dahlén, Niclas Bjarnegård, Toste Länne, Fredrik H Nystrom, Carl J Östgren

**Affiliations:** 1 Department of Medical and Health Sciences, Division of Community Medicine, Linköping University, Linköping SE-581 83, Sweden; 2 Diabetes Research Centre, Linköping University Hospital, Linköping, Sweden

**Keywords:** Abdominal obesity, Type 2 diabetes, Atherosclerosis, Intima-media thickness, Pulse wave velocity

## Abstract

**Background:**

Anthropometric measurements are useful in clinical practice since they are non-invasive and cheap. Previous studies suggest that sagittal abdominal diameter (SAD) may be a better measure of visceral fat depots. The aim of this study was to prospectively explore and compare how laboratory and anthropometric risk markers predicted subclinical organ damage in 255 patients, with type 2 diabetes, after four years.

**Methods:**

Baseline investigations were performed in 2006 and were repeated at follow-up in 2010. Carotid intima-media thickness (IMT) was evaluated by ultrasonography and aortic pulse wave velocity (PWV) was measured with applanation tonometry over the carotid and femoral arteries at baseline and at follow-up in a cohort of subjects with type 2 diabetes aged 55–65 years old.

**Results:**

There were significant correlations between apolipoprotein B (apoB) (r = 0.144, p = 0.03), C - reactive protein (CRP) (r = 0.172, p = 0.009) at baseline and IMT measured at follow-up. After adjustment for sex, age, treatment with statins and Hba1c, the associations remained statistically significant. HbA1c, total cholesterol or LDL-cholesterol did not correlate to IMT at follow-up. Baseline body mass index (BMI) (r = 0.130, p = 0.049), waist circumference (WC) (r = 0.147, p = 0.027) and sagittal Abdominal Diameter (SAD) (r = 0.184, p = 0.007) correlated to PWV at follow-up. Challenged with sex, SBP and HbA1c, the association between SAD, not WC nor BMI, and PWV remained statistically significant (p = 0.036). In a stepwise linear regression, entering both SAD and WC, the association between SAD and PWV was stronger than the association between WC and PWV.

**Conclusions:**

We conclude that apoB and CRP, but not LDL-cholesterol predicted subclinical atherosclerosis. Furthermore, SAD was more independent in predicting arterial stiffness over time, compared with WC, in middle-aged men and women with type 2 diabetes.

## Background

Anthropometric measures are useful in clinical practice since they are both non-invasive and cheap. Waist circumference (WC) is currently the most commonly used measurement for abdominal obesity, and highly associated with the risk of developing CVD [1]. However, recent studies suggest sagittal abdominal diameter (SAD) to be a better measurement to assess an adverse metabolic profile [2,3]. SAD has been shown to be strongly related to cardiovascular risk [4,5] and mortality [6,7]. Cardiovascular disease (CVD) is the major cause of morbidity and mortality in patients with type 2 diabetes. Traditional risk factors such as high LDL cholesterol and low HDL cholesterol, hypertension and smoking do not fully explain the increased cardiovascular risk in patients with type 2 diabetes [8]. Therefore, it is of great importance to identify better and non-invasive risk factor assessment tools to predict and ultimately to prevent CVD in this group.

Furthermore abdominal obesity is associated with increased levels of inflammatory markers [9]. Low- grade inflammation is involved in the atherosclerotic process and it has previously been shown that subjects with type 2 diabetes present with higher levels of inflammatory markers compared to subjects without diabetes [10]. Systemic low grade inflammation can be measured as circulating levels of the acute phase reactant C-Reactive Protein (CRP) [11].

Intima media thickness (IMT) of the carotid arteries measured by B-mode ultrasound is a well known, non-invasive marker of subclinical atherosclerosis [12,13]. While Pulse wave velocity (PWV) measured by tonometry provides a non-invasive estimate of arterial stiffness and is an independent predictive risk factor for all-cause mortality and cardiovascular mortality [14-16].

The aim of this study was to prospectively explore how laboratory and anthropometric risk factors predicted subclinical organ damage and, more specifically, compare SAD and WC as risk markers for subclinical, vascular organ damage, in a cohort of people with type 2 diabetes. A second aim was to explore the impact, over time, from systemic low-grade inflammation and lipids on arterial stiffness and atherosclerosis.

## Methods

### CARDIPP

We analyzed baseline data from the first 255 patients, who participated in a community-based cohort study, CARDIPP (Cardiovascular Risk factors in Patients with Diabetes – a Prospective study in Primary care). CARDIPP was launched in November 2005 and completed in 2008 with the aim to identify markers of cardiovascular disease to facilitate earlier and individually adjusted intervention, in middle aged men and women with type 2 diabetes. CARDIPP comprises data on an extended annual follow up on patients with type 2 diabetes, aged 55–66 years, consecutively recruited from 25 different primary health care centers in the counties of Östergötland and Jönköping, Sweden. All patients with type 2 diabetes, aged 55–66 years, who attended an annual follow-up at the health care centers, were consecutively invited to participate in the study. Detailed information about the structure and results from CARDIPP has been described previously [17,18]. The centres were located in different demographic areas and differed in size. However, the model of treatment and care of type 2 diabetes was organized similarly and all primary care centres adhered to the same national guidelines of diabetes care.

### Anthropometric measurements

Nurses especially dedicated to treatment of diabetes at the primary health care centers, measured height (to the nearest cm) and weight (to the nearest 0.1 kg) with the patients wearing light indoor clothing. Waist circumference (WC) was measured according to WHO’s recommendations with the patient standing, after a regular expiration, to the nearest cm, midway between the lowest rib and the iliac crest. SAD was recorded with the patient in the supine position and with bent knees, with a standardized sliding beam calliper at the highest point of the abdomen.

A standardized medical history was taken, including data on diabetes duration and ongoing medication.

### Laboratory tests

Blood specimens were drawn in the morning after a 10 hour over night fast. Routine tests such as HbA1c, plasma glucose and serum lipids were analysed according to routines at the primary health care centres. Levels of cholesterol, HDL and triglycerides was measured with enzymatic methodology and spectrophotometry, Selectra E,Vital Scientefic, Netherlands/Triolab. LDL was calculated by Friedewalds formula: LDL = cholesterol - HDL - 0.45 × fS/P-triglycerides.

HbA1c was analysed according to the Swedish Mono-S HPLC. In this study, all HbA1c values were converted to DCCT standard values using the formula: HbA1c DCCT = 0.923 × HbA1c (Mono-S) + 1.345 (R^2^ = 0.998) [19] and data on HbA1c is also reported in IFCC units. Blood samples were frozen for later analysis of CRP, apolipoprotein B (apoB) and apolipoprotein A-I (apoA-I) at the Centre for Laboratory medicine, Linköping University Hospital, Linköping, Sweden. Levels of apoB and apoA-I were measured by immunoturbidimetric assays, Advia 1800, DakoCytomation, Glostrup, Denmark. Coefficient of variation (CV) for apoB was 1.2% CV for apoA-I was 1.8%. CRP was also measured by immunoturbidimetric assays, Advia 1800, Siemens Diagnostic Medical Solutions, Erlangen, Germany. The detection level was 0.12 mg/L and CV was 1.6%. CRP values above 10mg/ml were excluded from the analyses according to current guidelines [20].

### Physiological vascular examinations

The blood pressure measurements, carotid ultrasonographic investigations, PWV and SAD were performed at the Department of Physiology, Linköping University Hospital, Linköping, Sweden and at the County Hospital Ryhov, Jönköping, Sweden. Systolic blood pressure (SBP) was measured with oscillometric technique (Dinamap PRO 200 Monitor, Critikon, Tampa, FL, USA) on the same occasion as the clinic physiological investigations. After at least 10 minutes rest in supine position, using an appropriately sized cuff, the BP was recorded in both arms. The mean value of two measurements in the arm with the highest systolic BP was used in further analyses in the study. IMT of the carotid arteries were evaluated using a B-mode ultrasound. A digital ultrasound system (ATL HDI 5000, Bothell, WA, USA) equipped with a broadband linear transducer (L12-5) was used for scanning the carotid artery in longitudinal section. ECG leads were connected. For lumen diameter (LD) and IMT determination, during diastole, three consecutive frozen images with special focus on lumen-intima echo and media-adventitia echo of the far arterial wall were saved for later analysis. The digital B-mode images were subsequently transferred to a PC, where software for off-line measurement of LD and IMT is installed (Artery Measurement System II, Image and Data Analysis, Gothenburg, Sweden). Calibration and subsequent measurement was performed by manually tracing a cursor along the leading edge of the intima-lumen echo of the near wall, leading edge of the lumen- intima echo and media-adventitia echo of the far wall. A 10 mm long section of the common carotid artery in the proximity of the carotid bulb was selected to obtain mean LD and far wall IMT. During analysis, the measurement window was hidden for the reader and values were saved in a text file. Mean values of IMT and carotid LD from both the right and the left sides were used in all analyses. Aortic PWV was measured with applanation tonometry (SphygmoCor® system, model MM3, AtCor Medical, Sydney, Australia) over the carotid and femoral arteries. The aortic pulse wave transit times were measured by electrocardiogram-guided readings of the femoral arterial pulse waves, using the carotid arterial pulse wave as the reference site. The surface distances were estimated from the suprasternal notch to the carotid and femoral measurement sites, respectively. PWV was calculated by dividing the surface distance with the pulse wave transit time yielding m × s^-1^.

### CARDIPP-Revisited

The CARDIPP-R comprises a re-investigation of the cohort four years after the completion of the baseline examination. In CARDIPP-R, all participants from the baseline study were invited to the re-investigation that was conducted at the Department of Physiology, Linköping University Hospital and at County Hospital Ryhov, Jönköping, Sweden. This study population comprises subjects with type 2 diabetes who were subjected to the baseline examination in 2006 and underwent the subsequent re-investigation in 2010.

The CARDIPP-R study protocol for the blood pressure measurements, the carotid ultrasonographic investigations and tonometry for measurements of the carotid, femoral and radial pulse pressure wave form and pulse wave velocity followed identical manual as in the baseline investigation. The routine laboratory tests were performed at the different health-care centers as described in the baseline protocol. In the follow-up investigation, CRP was not measured with an identical method as at baseline, thus our results regarding CRP values are confined to baseline data only.

### Statistics

IBM SPSS statistics 19 (IBM corporation, Somers, NY, USA) was used for statistical analyses. CRP, was log transformed due to skewed distribution. Pearson correlation coefficients were calculated between the different measurements, using bivariate correlation analysis. Statistical significance was assumed when p < 0.05. In multiple linear regression analyses with IMT or PWV, as dependent variables, the increase of one unit for each of the variables explored, confered a change in IMT or PWV respectively expressed as the regression coefficient (beta) with 95 percent confidence intervals (CI). In stepwise linear regression criteria for entry were p < 0.05 and for removal p > 0.1.

### Ethics

All participants gave written informed consent prior to participating in the study. The study, which complied with the declaration of Helsinki, was approved by the Regional Ethical Review Board in Linköping, Sweden.

## Results

**Figure 1:**
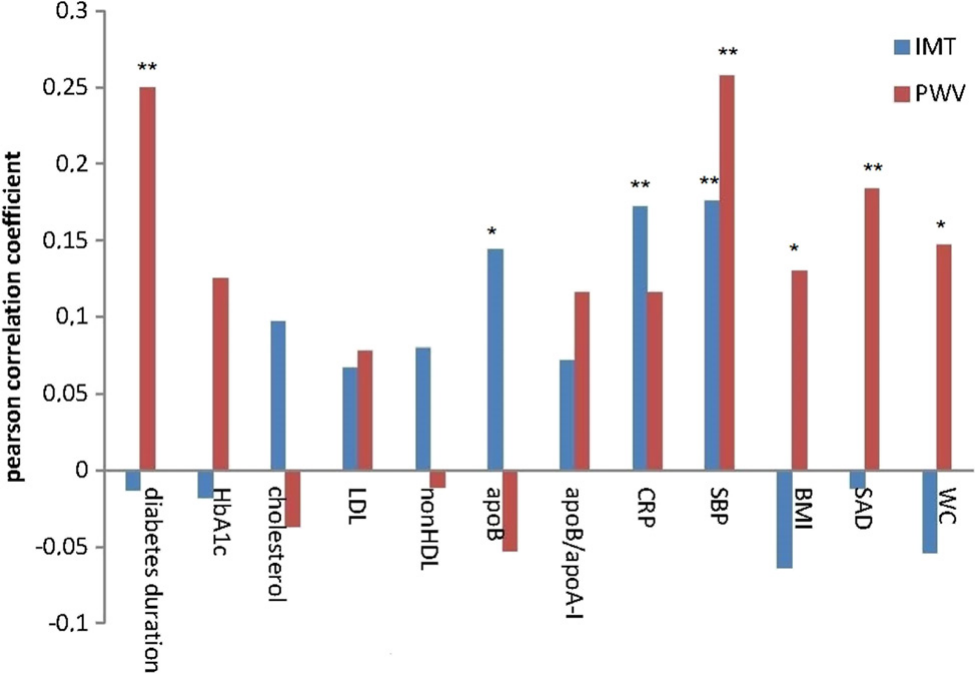
**Correlation between baseline characteristics 2006 and IMT and PWV 2010.** LDL; low-density lipoprotein, nonHDL; non high-density lipoprotein, apoB; apolipoprotein B, CRP; c-reactive protein, SBP; systolic blood pressure, BMI; body mass index; SAD; sagittal abdominal diameter, WC; waist circumference. * Correlation significant at p < 0.05, ** correlation significant at p < 0.01.

**Figure 2:**
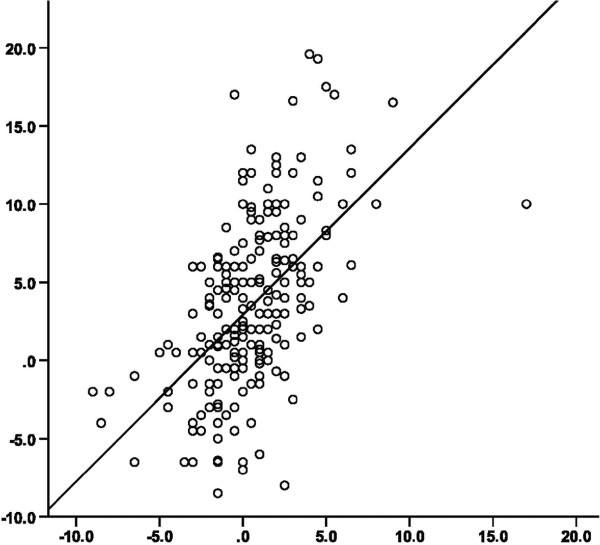
Relation between changes in SAD (x-axis) and WC (y-axis) after 4 years from the baseline investigation both measured in cm.

At the baseline investigation 64, (25%) patients were treated with diet and exercise only and the remaining patients were treated with oral glucose-lowering agents, n = 97, (38%), or insulin alone or in combination with oral glucose-lowering agents, n = 94, (37%). 121 (48%) patients were treated with statins and 164 (64%) patients reported treatment with antihypertensive agents. At the baseline investigation 2 subjects (0.8%) reported previous stroke and 23 (9%) subjects reported previous myocardial infarction. Table 1 displays the characteristics of the 255 subjects according to gender, at baseline investigation 2006 and at follow-up 2010. Figure 1 visualizes Pearson correlation coefficients between baseline variables and PWV and IMT measured at follow-up four years later. There were significant correlations between IMT and; apoB, CRP and systolic blood pressure (SBP) and there were significant correlations between PWV and; diabetes duration, SBP, BMI, WC and SAD. In Table 2, SBP, apoB and CRP that came out as correlated to IMT in Figure 1, remained statistically significant associated to IMT when further challenged in multiple linear regression analyses with IMT as dependent variable adjusted for baseline age, sex, treatment with statins, HbA1c. In Table 3, the associations between SBP, BMI, WC and SAD to PWV, were further challenged with sex, SBP and HbA1c. In these analyses the association between SAD, but not WC or BMI, and PWV remained statistically significant. In a stepwise linear regression, not in Table, entering both SAD and WC, the association between SAD at baseline and PWV at follow-up, was stronger than the association between WC and PWV. In Figure 2 the relation between the change (Δ) in WC and SAD is shown in a simple scatterplot. Δ WC shows greater variability compared to Δ SAD.

**Table 1:** Characteristics at baseline investigation (2006) and follow-up four years later (2010), in 172 men and 83 women with type 2 diabetes

		** Baseline**			**Follow-up**		
	**All**	**Men**	**Women**	**All**	**Men**	**Women**	**Δ All**
**Characteristics**	***mean (sd)***	***mean (sd)***	***mean (sd)***	***mean (sd)***	***mean (sd)***	***mean (sd)***	***mean (sd)***
Age (years)	61 (2.8)	61 (2.9)	61 (2.6)	65 (2.9)	65 (2.9)	65 (2.7)	4
BMI (kg/m^2^)	29.6 (5.0)	29.3 (4.7)	30.2 (5.5)	29.8 (5.1)	29.4 (5.0)	30.6 (5.3)	0.2 (2.2)
Sagittal Abdominal Diameter (cm)	25.2 (4.1)	25.4 (4.1)	24.8 (4.0)	25.9 (4.5)	26.0 (4.6)	25.8 (4.3)	0.6 (2.9)*
Waist circumference (cm)	102.4 (12.5)	103.2 (12.3)	100.5 (13.3)	105.6 (12.4)	106.1 (12.4)	104.5 (12.6)	3.3 (6.6)*
Systolic Bloodpressure (mmHg)	131 (17)	132 (16)	130 (17)	131 (16)	131 (15)	132 (21)	0.3 (17)
Diastolic Bloodpressure (mmHg)	75 (9)	77 (8)	70 (8)	73 (9)	75 (21)	69 (9)	−1.7 (8.5)*
HbA1c (% units)	7.0 (1.0)	7.0 (1.0)	7.0 (1.0)	7.2 (0.9)	7.2 (0.9)	7.3 (0.8)	0.23 (1.0)*
HbA1c (mmol/mol)	53.2 (11.6)	52.9 (11.6)	53.7 (11.8)	56.1 (9.8)	55.5 (10.2)	57.2 (8.9)	2.6 (10.9)*
Total cholesterol (mmol/L)	4.7 (1.0)	4.6 (0.9)	5.0 (1.0)	4.4 (1.0)	4.3 (1.0)	4.4 (0.9)	−0.4 (1.1)*
LDL cholesterol (mmol/L	2.6 (0.8)	2.6 (0.6)	2.3 (0.9)	2.4 (0.8)	2.4 (0.9)	2.3 (0.7)	−0.3 (0.9)*
HDL cholesterol (mmol/L)	1.4 (0.3)	1.3 (0.3)	1.5 (0.3)	1.2 (0.3)	1.2 (0.3)	1.3 (0.4)	−0.1 (0.2)*
Non HDL cholesterol (mmol/L)	3.4 (1.0)	3.3 (0.9)	3.6 (1.1)	3.1 (0.9)	3.1 (0.9)	3.1 (0.9)	−0.3 (1.1)*
ApoB/ApoA-I	0.73 (0.17)	0.73 (0.16)	0.71 (0.19)	0.66 (0.20)	0.68 (0.20)	0.61 (0.18)	−0.08 (0.18)*
ApoB	0.95 (0.19)	0.94 (0.18)	0.97 (0.20)	0.88 (0.21)	0.89 (0.21)	0.86 (0.21)	−0.08 (0.21)*
Serum Triglycerides (mmol/L)**	1.5 (6.5)	1.4 (6.4)	1.5 (4.4)	1.4 (6.0)	1.4 (6.0)	1.5 (3.4)	−0.09 (7.5)
Pulse wave velocity (m/s)	10.2 (2.2)	10.3 (2.2)	10.0 (2.2)	11.0 (2.4)	11.1 (2.4)	11.0 (2.5)	0.84 (1.9)*
Intima-Media Thickness (mm)	0.70 (0.17)	0.70 (0.18)	0.67 (0.14)	0.78 (0.20)	0.79 (0.23)	0.77 (0.15)	0.09 (0.2)*
Treatment with statins n (%)	121 (47.5%)	81 (47%)	40 (48.2%)	175 (69%)	111 (61%)	64 (78%)	54 (21%)*
Smoking n (%)	41 (16%)	24 (14%)	17 (20.5%)	35 (14%)	19 (11%)	16 (19.5%)	−6 (−2%)*

* Difference at p < 0.01 level ** Data on variables with skewed distribution are given with median and interquartile range.

**Table 2:** Associations between IMT and baseline levels of CRP, apoB, and systolic BP as independent variables one by one in different settings

		**IMT**		
**Variable**	**(Unit)**	**Beta coefficient**	**95% CI**	***P***
CRP	(mg L^-1^)	0.069	(0.013-0.125)	0.016
apoB	(g L^-1^)	0.173	(0.015-0.331)	0.032
Systolic BP	(mmHg)	0.002	(0.000-0.004)	0.026

Multiple linear regression analyses of IMT measured 2010 as dependent variable adjusted for age, sex, treatment with statins, HbA1c measured at baseline and PWV. The increase of one-unit for each of the variables confers a change in IMT mm, expressed as the regression coefficient (Beta) with 95 percent CI.

**Table 3:** Associations between PWV and SBP, BMI, SAD and WC as independent variables one by one in different settings

**PWV**				
**Variable**	**(Unit)**	**Beta coefficient**	**95% CI**	***p***
SBP*	(mmHg)	0.034	(0.015-0.053)	<0.001
BMI	(kg m^2^)	0.046	(−0.022-0.114)	0.181
SAD	(cm)	0.092	(0.006-0.179)	0.036
WC	(cm)	0.020	(−0.007-0.048)	0.143

Multiple linear regression analyses of PWV measured 2010, as dependent variable adjusted for, sex, HbA1c and systolic blood pressure. The increase of one-unit for each of the variables confers a change in PWV m s^-1^, expressed as the regression coefficient (Beta) with 95 percent CI. * not adjusted for SBP.

## Discussion

To the best of our knowledge this is the first prospective study comparing SAD and WC, exploring the associations between abdominal obesity and arterial stiffness measured as PWV, in men and women with type 2 diabetes. We report that SAD was a more independent predictor for increased arterial stiffness over time compared with WC. Previous studies have shown that SAD is a predictor of insulin resistance and levels of CRP in immigrant women from the middle-east [2] as well as in obese men [3] and that SAD is closely related to visceral adiposity [21,22]. In a recent, prospective study of a Finnish population SAD was more predictive of incident diabetes compared to WC [23]. Visceral fat is a potent source for expression of proinflammatory, atherogenic cytokines and is closely related to levels of inflammatory markers [24]. Recent studies have also shown that reducing the visceral fat improves endothelial dysfunction, which is an early predictor for cardiovascular disease [25]. Persons with type 2 diabetes have an increased risk of all-cause mortality compared to a population without diabetes [26] and cardiovascular disease is the major cause of morbidity and mortality in these subjects.

The reliability of measurements is an important factor to consider in clinical practice. SAD has a high reliability in both lean and obese subjects [27]. WC can be measured in various ways and there is no consensus about which is the best measurement protocol [28]. In this study we have chosen the method of measuring WC recommended by WHO, where WC is measured midway between the last rib and the iliac crest and not at maximum WC, compared to SAD which is measured at maximum abdominal height. This might partly explain our finding that SAD was more robustly associated to the change in arterial stiffness. SAD is a clinically feasible measurement with high reproducibility, which gives a good approximation of the atherogenic visceral fat. WC can be measured in variable ways, at the maximum waist or in the midline between the lower rib and iliac crest. Depending on the patient’s individual body composition the subcutaneous fat is added to the visceral fat when measuring waist and it could explain some of the differences compared to SAD. When measuring SAD the patient is in the supine position which allows the subcutaneous fat to redistribute along the sides of the abdomen, hence the height of the abdomen is more likely to provide information on the amount of visceral fat.

Traditional risk factors such as high LDL cholesterol and low HDL cholesterol, hypertension and smoking do not fully explain the increased cardiovascular risk in patients with type 2 diabetes [8]. Therefore, it is of great importance to identify and evaluate risk factor assessment tools to predict and ultimately to prevent CVD. ApoB is found in chylomicrons, VLDL, IDL and LDL particles. Since each of these particles contains one single apoB, measurement of apoB represents the total burden of the lipoproteins considered most atherogenic. Hence a consensus document from the American Diabetes association and the American College of Cardiology Foundation for lipoprotein management in patients with cardiometabolic risk, has suggested treatment goals for apoB [29]. In recent large Asian studies, apoB was a strong predictor of the metabolic syndrome [30] and was proven to be reliably estimated by routine lipid biochemistry [31]. In this numerically quite small observational prospective study we were able to confirm previous findings that apoB is superior to conventional lipids in predicting atherosclerotic related vascular damage [32]. Furthermore, in this study baseline levels of CRP predicted the development of subclinical atherosclerosis which may not come as a surprise since CRP is a well known predictor of CVD and also of type 2 diabetes [33].

The major limitation of this prospective study is the relatively small number of study subjects, which precludes subgroup analyses. Another limitation is that insulin resistance, which is an independent predictor for atherosclerosis and arterial stiffness [34], was not measured. Furthermore, the study-participants had multiple risk factors for cardiovascular disease, such as hypertension and dyslipidemia, and hence we cannot specify exact mechanistic progressive interactions of changes in the different mediators of vascular disease. In particular since these risk factors are affected by the medications for these conditions.

Thus, there is still a need for further prospective studies on larger cohorts comparing the clinical utility of the anthropometric measurements.

## Conclusions

In summary, we conclude that SAD, if confirmed in future, larger studies, may be used as a more independent risk-assessment tool compared with WC in clinical practice, to identify persons with type 2 diabetes at high cardiovascular risk. Furthermore the superiority of apoB over LDL-cholesterol as predictor of subclinical atherosclerosis, is in line with previous observational studies [35,36].

## Abbreviations

SAD: Sagittal abdominal diameter; IMT: Carotid intima- media thickness; PWV: Pulse wave velocity; apoB: ApolipoproteinB; apoA-I: Apolipoprotein A-I; CRP: High sensitive c-reactive protein; LDL: Low density lipoprotein; HDL: High density lipoprotein; VLDL: Very low density lipoprotein; IDL: Intermediate density lipoprotein; BMI: Body mass index; WC: Waist circumference; SBP: Systolic blood pressure; DBP: Diastolic blood pressure; CVD: Cardiovascular disease; CV: Coefficient of variation; CARDIPP: Cardiovascular risk factors in patients with diabetes – a prospective study in primary care.

## Competing interests

The authors declare no conflicts of interests.

## Authors’ contributions

CJÖ, FN and TL designed and lead the study. NB Performed physiological vascular examinations and ED performed the statistical analyses. All authors contributed in writing the manuscript and all authors read and approved the final version of the manuscript.
